# The Perceived Contribution of Older People to Climate Change Impact, Mitigation, and Adaptation: Measurement Development and Validation

**DOI:** 10.1093/geroni/igad095

**Published:** 2023-09-09

**Authors:** Liat Ayalon, Senjooti Roy

**Affiliations:** Louis and Gabi Weisfeld School of Social Work, Bar Ilan University, Ramat Gan, Israel; Louis and Gabi Weisfeld School of Social Work, Bar Ilan University, Ramat Gan, Israel

**Keywords:** Ageism, Intergenerational relations, Climate change, Measurement development

## Abstract

**Background and Objectives:**

To improve the understanding of ageism toward older people in the context of climate change, the present study developed and validated a new measure that examines the perceived negative and positive contributions of older people to climate change impact, mitigation, and adaptation efforts.

**Research Design and Methods:**

Four studies (*N* = 774) were conducted to develop a new measure and evaluate its reliability and validity, relying on exploratory factor analysis, reliability analysis, confirmatory factor analysis, multiple-group analysis (Australia and India), discriminative validity, and convergent and divergent validity.

**Results:**

A 2-subscale measure covering older people’s perceived negative contribution to climate change effects (5 items) and perceived positive contribution to adaptation and mitigation measures (3 items; eg, negative, and positive ageism in the context of climate change) was supported by the data. The measure has demonstrated adequate validity and reliability.

**Discussion and Implications:**

The measure highlights a relatively neglected area in current climate change discourse and may assist in identifying ways to improve intergenerational solidarity as part of climate change adaptation and mitigation efforts toward building a world for all ages under a healthy climate, which allows for healthy aging and healthy longevity. These objectives are in line with the current mission posed by the UN Decade of Healthy Ageing.

**Translational Significance:** The study highlights the significance of intergenerational relations in the context of climate change. Intergenerational relations have both positive and negative aspects. The new measure can be used in further research on the topic.

Climate change has real-life implications for the life of each and every one of us. It has been shown to impact our human rights, including access to water, food, livelihood, and shelter. In the case of older people, the effects of climate change are particularly deleterious (Human Rights Council, 2021). Research has shown that older people exposed to extreme climate change events are vulnerable both mentally and physically (Ayalon et al., 2021). Specifically, older people are more susceptible to cardiovascular events, kidney diseases, and urinary tract infections following exposure to severe heat (Borg et al., 2019; Kenney et al., 2014). Exposure to air pollution has been associated with a higher incidence of dementia (Peters et al., 2019). The impact of climate change on older people is not only physiological in nature. Guilt, depression, suicide ideation, and substance use are some of the emotional consequences of exposure to extreme weather events (Ayalon et al., 2021; Charlson et al., 2021). Research also has shown that extreme weather events place older people at a greater risk for developing posttraumatic stress disorder compared with younger people (Kun et al., 2013). Moreover, older people have a greater mortality risk following extreme weather events and evacuation efforts (Aida et al., 2017; Åström et al., 2011; Willoughby et al., 2017).

The increased susceptibility of older people to extreme weather events, however, should not be attributed solely to their advanced ages and corresponding physiological states. In fact, research has shown that social support plays a major role in people’s ability to survive climate events (Klinenberg, 2015), highlighting the role of social capital in adaptation to climate change (Durant, 2011). Moreover, the neglect of older people in climate policy (Human Rights Council, 2021) and the failure of social, political, and economic institutions to ensure their safety further exacerbates the situation (Ayalon et al., 2021; Dwyer et al., 2004). Importantly, it often is not age alone, but age in intersection with other attributes, such as gender, socioeconomic status, mental health conditions, and physical or cognitive functioning, which make older people particularly susceptible to climate change effects (Ayalon et al., 2021; McDermott-Levy et al., 2019; World Health Organization, 2014).

Although acknowledging older people’s susceptibility, it also is important to acknowledge their responsibility for the current climate situation. The few studies that addressed this topic have identified several explanations for the lack of engagement in pro-environmental behaviors among older people. Lack of knowledge, disbelief about the negative forecasts associated with the changing climate, and a belief that “science will save us” or that “a single person cannot make a difference” also serve as barriers to pro-environmental behaviors among older people (Moody, 2014; Moody, 2017). Likewise, older people also are less likely to believe that climate change is real or is a result of human action (Milfont et al., 2021). Moreover, a qualitative study of older people has found that the majority do not engage in climate activism and tend to believe that the responsibility to act is on governments. Older people in that study also engaged in very restricted pro-environmental behaviors (Ayalon et al., 2022b). Furthermore, the carbon footprint of older people is larger not only because they have lived for a longer period but also when compared to younger people within a single point in time (Estiri & Zagheni, 2019). Hence, there is some truth to the accusations that older people have disproportionately contributed to the current climate situation.

## Intergenerational Relations and Climate Change

Theories of social justice provide a useful framework to explore the role of age and generation in climate discourse. Social justice refers to the equal and equitable distribution of wealth, opportunities, and power among different groups (Borras Jr. & Franco, 2018; Walster & Walster, 1975). It also refers to the acknowledgment and recognition of inequalities among different groups (Benjaminsen et al., 2021). In the case of climate change, social justice addresses mitigation and adaptation efforts, which consider the differential vulnerabilities, resources, and interests of different social groups (Baxi, 2016).

Social justice has relevance to intergenerational relations and climate change (Page, 1999). This is because both the impact of climate change as well as the impact of mitigation and adaptation efforts have a temporal dimension. (In)action of the present or the past is likely to have relevance even for those who are not yet born and certainly for current generations of children and youth. According to the Intergovernmental Panel on Climate Change (IPCC, 2021), climate extremes are directly attributable to human activities. Some of the effects of climate change on humans and nature are beyond adaptation and thus, already represent irreversible progressive effects. This will result in differential impacts of climate change on certain groups in society over time (Intergovernmental Panel on Climate Change, 2021).

Both younger and older people are susceptible to the negative impact of climate change. Older people are more likely to develop medical conditions, mental health conditions, and increased mortality following climate events (Filiberto et al., 2009). However, younger people are susceptible not only physiologically but also because they are likely to experience the negative impact of climate change for a longer period of their lives (Sanson & Burke, 2020). At the same time, it is older people who are asked to make compromises for a future of which they will not be part. Hence, social justice in the context of intergenerational relations refers to the unequal distribution of resources and burdens as well as the recognition of such inequalities across generations (Page, 1999).

Social justice is relevant not only from a distributional perspective but also from the recognition perspective (Benjaminsen et al., 2021). This concerns the social recognition of those most impacted by climate change as well as those affected by mitigation and adaptation efforts associated with climate change. It is younger people who often feel as if their voice is not heard. They do not have voting power and are unable to influence political decisions that concern their own future (Han & Ahn, 2020). Older people, on the other hand, hold political power, yet there is a limited societal acknowledgment of their susceptibility to climate change effects (Ayalon et al., 2021). Additionally, they are held responsible for their lack of urgency in addressing climate change.

A recent scoping review on intergenerational relations in the context of climate change has found that scientific literature addresses intergenerational conflict and tension on the one hand and solidarity and transmission of knowledge on the other (Ayalon et al., 2022a). The study concluded that there is a shortage of validated and reliable measures to assess areas of conflict and solidarity between generations in the context of climate change (Ayalon et al., 2022a). The concept of intergenerational ambivalence (Lüscher & Pillemer, 1998), the co-occurrence of conflict and solidarity, was identified as particularly relevant to describe climate change discourse (Ayalon et al., 2022a). On the one hand, there are areas of tension and conflict between generations who may view the climate change experience as socially unjust given the disproportional contribution of older people to the current situation and the deprivation of younger people of political power; on the other hand, there are instances of solidarity between generations which are highly evident in the collective efforts of different age groups aimed toward fighting climate change (Ayalon et al. 2022a). Additionally, older people can serve as active advocates for the use of sustainable energies; they can benefit from participating in the climate change movement and many are often willing to make extensive sacrifices in order to ensure a healthy climate for future generations (Pillemer & Filiberto, 2017; Pillemer et al., 2009).

### Ageism and Climate Change

Ageism, defined as stereotypes, prejudice, and discrimination toward people because of their age (Iversen et al., 2009) is one driver of intergenerational relations (Ayalon, 2020). Although ageism can be directed toward both young and old (Ayalon & Tesch-Römer, 2018), the present focus is on ageism toward older people. Ageism has been shown to have detrimental impacts on the health and well-being of older people (Chang et al., 2020). It is highly prevalent, being reported by one in two people, globally (Officer et al., 2020) and experienced by one in three Europeans (Ayalon, 2014). A recent systematic review has pointed to a shortage of valid and reliable measures of ageism, with a particular need to focus on context-specific measures, given the fact that ageism is manifested differently in different contexts (Ayalon et al., 2019).

Considering current calls to develop psychometrically sound measures of ageism, and the increasing tension between generations in the context of climate change brought on by ageist stereotypes, the present study describes the development of a new measure to assess the two poles of ageism toward older people as manifested in intergenerational relations in the context of climate change. The development of a measure that captures both conflict and solidarity is a first step toward enhancing the understanding of intergenerational relations in the context of climate change and the possible impact of ageism directed toward older people. To develop the measure, we conducted four studies with three different samples (*N* = 774), from two countries (Australia and India), recruited online between May 2021 and February 2022. In Study 1, we conducted an exploratory factor analysis (EFA) to select appropriate items for the new scale. In Study 2, we recruited two additional samples to establish confirmatory factor analysis (CFA) and cross-cultural validity. In Study 3, we established discriminative validity by examining known-subgroup differences. In Study 4, we examined divergent and convergent validity by examining the correlation of the new measure with already established measures of ageism and death anxiety. We selected Australia and India for this study due to their recent experiences with severe climate events. Although the two countries are culturally very different, we selected them to examine how common climate events such as floods and record-breaking heat may impact the perceptions of the populations of the two countries. Because this study specifically addresses the subject of ageism towards older adults, we collected data from all adult age groups to identify similarities and differences in how they view older generations in relation to climate change.

## Study 1: Item Selection and Exploratory Factor Analysis

### Objective

The goal of this study was to select items for the new scale, relying on EFA.

### Item Generation

Eighteen potential scale items were derived based on (a) a scoping review of the literature (Ayalon et al., 2022a); (b) interviews with climate activists (Roy & Ayalon, 2023); (c) a review of statements made by prominent climate activists in media (Roy & Ayalon, 2022) and (d) open-ended responses of laypeople to a question about the role of age in climate change effects and mitigation and adaptation efforts. Items were formed based on thematic analysis (Braun & Clarke, 2012) of these varied sources of information. Items addressed the role of older people in climate change effects as well as mitigation and adaptation efforts. Both positive and negative statements about older people’s contribution to and impact of climate change, including involvement in mitigation and adaptation efforts were presented. Selected items were reviewed by six experts in the field of gerontology for face validity and were modified accordingly. When selecting the items, we specifically aimed to address two poles: (a) negative ageist stereotypes, defined as unfavorable perceptions regarding older people’s contribution to climate change and lack of involvement in mitigation efforts, and (b) positive ageist stereotypes, defined as acknowledgment and appreciation of older people’s contribution to adaptation and mitigation efforts. Items were selected to ensure their relevance, comprehensiveness, and comprehensibility (Gagnier et al., 2021). See Supplementary Material for a list of proposed items. In our study, individuals over the age of 60 were categorized as the older generation.

### Participants

In total, 250 Australians participated in this study (Sample 1). The average age was 46.20 (*SD* = 18.67) and 52.4% were women. The majority were Christian (46.0%) with undergraduate education (45.6%).

### Procedure

All studies received the ethical approval of the authors’ institution’s ethics committee. All participants signed an online informed consent prior to their participation. Respondents were recruited via an Australian survey agency in May 2021. Respondents received financial compensation for their participation. They were asked to rank the 18 items on a scale between 1-strongly disagree and 7-strongly agree. The instruction form stated that individuals over the age of 60 were categorized as the older generation.

### Analysis

Using SPSS version 27 (IBM, 2017), we conducted an EFA to examine intercorrelations among items. We relied on principal component analysis with varimax rotation. Double-loading items and those loading lower than 0.4 on their respective factor were eliminated. We also calculated the internal consistency of the final scale and subscales.

### Results

EFA resulted in a three-factor solution (available upon request). However, after eliminating items with cross loadings or loading lower than 0.4, eight items were maintained, representing a two-factor solution corresponding to the perceived negative versus positive contribution of older people to climate change impact as well as adaptation and mitigation efforts, thus, broadly reflecting negative and positive ageism. Five items represented perceived negative contribution, whereas three items covered perceived positive contribution. All items showed moderate-strong loadings on the two-factor solution. Cronbach’s alpha was high-moderate for the two subscales and moderate for the overall scale (0.91, 0.72, 0.76, respectively). Table 1 illustrates item loadings. Table 2 illustrates the descriptive characteristics of the eight items selected. There was a moderate floor effect for all five items under the negative contribution subscale (possibly indicating that people refrain from acknowledging ageist attitudes) and a ceiling effect for one item under the positive contribution subscale.

**Table 1.** Exploratory Factor Analysis-Factor Loadings Across the Different Studies

Item	S1 Australia (*N* = 250) Loading	S2 India (*N* = 274) Loading	S3 Australia (*N* = 250) Loading
Factor 1: Older people’s negative contribution (α)	0.91	0.86	0.89
Older people are living far too long and becoming a burden on the planet’s resources.	0.84	0.83	0.79
Older people’s maximum benefit to society would be to reduce their carbon footprint by withdrawing from active life after retirement.	0.85	0.80	0.82
Older people are selfish about accumulating wealth even though it harms the planet and the future of younger generations.	0.89	0.82	0.86
Older people have limited will to work for the betterment of the planet.	0.83	0.81	0.82
Older people have too much voting power on issues like climate change.	0.88	0.72	0.84
Factor 2: Older people’s positive contribution (α)	0.72	0.76	0.61
Older people can be powerful allies in the fight against climate change.	0.66	0.77	0.47
Many older leaders use their office to fight climate change for the benefit of future generations.	0.86	0.84	0.85
Older people can be trusted to vote for political candidates who support the fight against climate change.	0.86	0.84	0.85
Total score (α)	0.76	0.78	0.71
%Variance explained	70.52%	65.58%	65.23%

*Note*: S = sample.

**Table 2.** Mean, Standard Deviation, and Range of Items (*N* = 250; S1)

Item	Mean (*SD*)	Item score (%)						
		1	2	3	4	5	6	7
1.Older people are living far too long and becoming a burden on the planet’s resources.	3.24 (1.92)	27.6	15.6	9.2	21.2	11.6	8.4	6.4
2.Older people’s maximum benefit to society would be to reduce their carbon footprint by withdrawing from active life after retirement.	2.96 (1.82)	34.3	11.6	11.2	24.0	8.8	5.2	4.8
3.Older people are selfish about accumulating wealth even though it harms the planet and the future of younger generations.	3.28 (1.92)	28.4	12.0	9.2	25.6	10.0	7.6	7.2
4.Older people have limited will to work for the betterment of the planet.	3.55 (1.77)	16.8	16.9	14.0	23.2	13.6	11.6	4.8
5.Older people have too much voting power on issues like climate change.	3.42 (1.84)	22.7	13.5	12.0	21.2	16.4	8.4	5.6
***6.*** Older people can be powerful allies in the fight against climate change.	5.42 (1.32)	1.6	8.0	3.2	19.6	22.8	27.2	24.8
7.Many older leaders use their office to fight climate change for the benefit of future generations.	4.48 (1.44)	2.8	8.8	9.2	32.0	24.0	15.6	7.6
8.Older people can be trusted to vote for political candidates who support the fight against climate change.	4.49 (1.50)	4.8	4.4	10.0	35.6	18.8	16.0	10.4
Older people’s negative contribution	3.29 (1.59)							
Older people’s positive contribution	4.77 (1.13)							

*Notes*: S = sample. Items range between 1 and 7; higher scores represent higher agreement.

## Study 2: Confirmatory Factor Analysis and Cross-Cultural Validity

### Objective

This study aimed to establish the structural validity of the new scale through CFA to support the two dimensions of the new scale. Cross-cultural validity was established by examining measurement invariance of the new scale in two different countries (Australia and India; Gagnier et al., 2021).

### Analysis

Using Mplus 8 (Muthén & Muthén, 1998–2011), we examined CFA of the two-factor model relying on three different samples: the original Australian sample reported in Study 1, an Indian sample (*N* = 274; Sample 2) and an additional Australian sample (*N* = 250; Sample 3). Model fit was determined by using the following fit indices: *X*^2^, Comparative Fit Index (CFI), Tucker–Lewis index (TLI), root mean square error of approximation (RMSEA; Taasoobshirazi & Wang, 2016). CFI with values of 0.90–0.95 indicates an acceptable fit and CFI > 0.95 indicates a good fit, TLI > 0.9 indicates an acceptable model fit and TLI > 0.95 indicates a good fit, RMSEA = <0.05 indicates a well-fitted model and values between 0.05 and 0.08, indicate moderate fit (Hoyle, 1995; Hu & Bentler, 1999).

To ensure cross-cultural validity, we examined measurement invariance across the two samples, using multi-group CFA (Milfont & Fischer, 2010). This was determined through a series of models, examining *configural* and *metric* invariance. We started out with a CFA analysis to examine the structural validity of the proposed two-factor solution. This model was fitted to each group separately, with loadings allowed to be free. This was done to test for *configural invariance*, which examines whether the same model is reproduced in each group. To examine *metric invariance*, we set factor loadings to be equal across groups. Metric invariance suggests that factor variance and covariance can be compared. In each step, we examined fit indices of constrained vs. non-constrained models. Invariance is determined when Δ*X*^2^ (Δ*df*) is non-significant, suggesting that the more restrictive and parsimonious model is not significantly worse than the one that allows for variations across groups.

### Participants

An online sample of 274 Indian people over the age of 18 was recruited between May and November 2021. The average age of respondents was 42.13 (*SD* = 17.69), 40.9% were women, and 49.1% had postgraduate education. For the second Australian sample, 250 respondents were recruited in February 2022. Their average age was 45.64 (*SD* = 19.64) and 51.4% were women. The majority were Christian (45%) with undergraduate education (38%).

### Procedure

For the Indian and Australian samples, respondents were recruited via an Indian and an Australian survey agency, respectively. Respondents received financial compensation for their participation. They were asked to rank the eight items on a scale between 1-strongly disagree and 7-strongly agree.

### Results

EFA resulted in a two-factor solution for the Indian and the Australian samples. Factor loadings and Cronbach’s Alpha are reported in Table 1. Two subscales emerged as in Study 1. Next, CFA was conducted for all three samples. Table 3 summarizes the fit indices for a single versus a two-factor solution across the three samples. Using the Hu and Bentler (1999) criteria, fit indices suggest a reasonable fit for the two-factor solution compared with the single-factor solution in all three samples.

**Table 3.** Confirmatory Factor Analysis Fit Indices for a Single- and a Two-Factor Solution

Goodness of fit index	S1 (250)	S2 (274)	S3 (250)
One-factor model			
*X*^2^_(*df*)_	229.808 (20)	274.740 (20)	190.183 (20)
CFI	0.793	0.690	0.802
TLI	0.710	0.567	0.723
RMSEA	0.205 (0.181–0.229)	0.215 (0.193–0.238)	0.184 (0.161–0.209)
Two-factor model			
*X*^2^_(*df*)_	70.105 (16)^1^	53.607 (18)^2^	58.38 (19)^3^
CFI	0.939	0.957	0.954
TLI	0.893	0.933	0.933
RMSEA	0.116 (0.089–0.145)	0.085 (0.059–0.111)	0.091 (0.065–0.118)

*Notes*: CFI = Comparative Fit Index; RMSEA = root mean square error of approximation; S = sample; TLI = Tucker–Lewis index.

^1^Residual covariances of items 1 & 2, 1 & 3, 2 & 3, 7 & 8 were allowed to correlate 2 residual covariances of items 1 & 5 were allowed to correlate 3 residual covariances of items 1 & 2 were allowed to correlate.

Next, we examined multiple-group CFA, gradually testing the level of invariance across the three samples. *Configural invariance* resulted in adequate fit indices, suggesting an invariant structure across the three samples. *Metric invariance* resulted in threshold significance at 0.001, suggesting that factor loadings are noncomparable. Thus, *scalar invariance* (item intercepts are equivalent across groups) was not pursued. See Table 4 for details.

**Table 4.** Cross-Cultural Validity—Multiple-Group Comparison

Invariance model	Restrictions	*X* ^2^(*df*)	ΔX^2^ (Δ*df*)	*p*	CFI	TLI	RMSEA
Configural invariance		243.586 (61)			0.932	0.907	0.108 (0.094–0.122)
Metric invariance	Factor loadings	273.228 (76)	29.642 (15)	0.01	0.927	0.919	0.100 (0.088–0.113)

*Notes*: CFI = Comparative Fit Index; RMSEA = root mean square error of approximation; TLI = Tucker–Lewis index.

## Study 3: Discriminative Validity

### Objective

This study examined subgroup differences on the new scale with regard to age and gender. This is expected to establish the discriminative validity of the new scale (Gagnier et al., 2021).

### Analysis

Discriminative validity was examined through age and gender subgroup comparisons. We expected to see age differences in response to the two subscales so that older respondents are more likely to point to positive contributions of older people to climate change mitigation and adaptation efforts and younger respondents are more likely to point to negative contributions. This is in line with past research showing higher levels of negative ageism reported by younger age groups (Rupp et al., 2005). We expected no gender differences in responses to the two subscales. This is because although ageism is thought to be more prevalent among women (Krekula et al., 2018), results concerning gender differences in reports of ageism are mixed (Bodner et al., 2012; Cherry et al., 2016; Kalavar, 2001; Rupp et al., 2005).

### Participants

Respondents from the three samples described earlier participated in this analysis, which was conducted separately per sample.

### Results

Table 5 summarizes the results. Across the three samples, younger participants were more likely to point to the negative contributions of older people and older respondents were more likely to point to positive contributions. Compared with men, women were more likely to point to negative contributions of older people to climate change in two of the three samples.

**Table 5.** Discriminative Validity: Gender and Age Differences in Perceived Positive and Negative Contribution

Factor	Total score	Men	Women	*t*-test(*df*)	*p*	Young < 45	Old ≥ 46	*t*-test(*df*)	*p*
S1 Australia (*N* = 250)									
Negative contributions	3.30 (1.59)	3.03 (1.47)	3.53 (1.68)	−2.45 (272)	0.01	3.96 (1.52)	2.58 (1.34)	7.52 (248)	<.001
Positive contributions	4.78 (1.13)	4.77 (1.16)	4.79 (1.12)	−1.6 (246)	0.84	4.59 (1.14)	4.98 (1.10)	−2.76 (248)	<.01
S2 India (*N* = 274)									
Negative contributions	3.96 (1.52)	3.77 (1.48)	4.23 (1.57)	−2.46 (272)	0.015	4.21 (1.57)	3.53 (1.36)	3.61 (273)	<.001
Positive contributions	5.43 (1.18)	5.50 (1.12)	5.28 (1.25)	1.53 (272)	0.12	5.22 (1.28)	5.72 (.92)	-3.28 (272)	<.001
S3 Australia (*N* = 250)									
Negative contributions	3.12 (1.46)	3.01 (1.40)	3.17 (1.49)	−.83 (246)	0.41	3.88 (1.31)	2.30 (1.55)	10.09 (248)	<.001
Positive contributions	4.52 (.91)	4.45 (.90)	4.60 (.91)	− 1.34 (246)	0.18	4.52 (.96)	4.86 (.98)	−2.75 (248)	<.01

*Note*: S = sample.

## Study 4: Convergent and Divergent Validity

### Objective

We examined the relationship of the newly developed measure with other constructs. We expected the two subscales to negatively correlate with each other as they measure two opposing sets of attitudes. Given the fact that the new measure addressed ageism in the context of climate change, we expected it to correlate with a different measure of ageism. Specifically, we expected the perceived negative contribution of older people to climate change subscale to correlate positively with the Succession, Consumption, Identity (SIC) measure of ageism (North & Fiske, 2013). We further expected the perceived positive contribution of older people to climate adaptation and mitigation efforts subscale to correlate negatively with this measure. We also expected fear of death (Carmel & Mutran, 1998) to be positively correlated with perceived negative contribution subscale and negatively correlated with the positive contribution subscale. This follows the terror management theory which posits that higher levels of death anxiety are associated with higher levels of negative ageism toward older people (Martens et al., 2005). This also is based on past research which has suggested an association between death anxiety and climate change concerns (Mann & Wolfe, 2016).

### Participants

The Australian sample recruited as part of Sample 3 participated in this study.

### Ageism

It was measured using the Succession, Consumption, Identity scale (North & Fiske, 2013). The scale consists of three factors of 20 items. The scale addresses prescriptive expectations concerning older people who should give the right of the way to younger people, not consume scarce resources, and maintain an appropriate age identity. Items are ranked on a 1–6 scale, with a higher score indicating greater ageism. Mean (*SD*) = 6.83(3.47), α = 0.92.

### Fear of Death

It was measured using six items taken from the fear of death and dying scale (Carmel & Mutran, 1998). Items are ranked on a five-point scale, with a higher score indicating greater fear. Mean (*SD*) = 5.96(4.38), α = 0.86.

### Results

There was no significant correlation between the two subscales (e.g., negative, and positive ageist stereotypes; *r* = −0.09), suggesting that the two subscales represent different constructs. As expected, higher levels of ageism were associated with higher levels of perceived negative contribution of older people (*r* = 0.64, *p* < .01) to climate change, and lower levels of perceived positive contribution of older people to climate action (*r* = −0.23, *p* < .01). Fear of death was positively correlated with the negative contribution subscale (*r* = 0.14, *p* < .01) but had no correlation with the perceived positive contribution subscale (*r* = −0.07).

## Discussion

Ageism toward older people is highly prevalent worldwide. It is manifested at the individual, micro-level; at the interpersonal, meso-level; and at the institutional, macro-level. Ageism is prevalent in a variety of settings and contexts (Ayalon & Tesch-Römer, 2018; World Health Organisation, 2021a). More recently, ageism was identified in the context of climate change discourse, and its potential negative effects on intergenerational relations have been pinpointed (Ayalon, 2020; Ayalon et al., 2022a).

In this paper, we describe the development and validation of a new measure to assess negative and positive ageism in the context of climate change. The measure consists of eight items, which cover both the perceived negative contribution of older people to climate change effects and the perceived positive contribution of older people to climate change mitigation and adaptation efforts. The perceived negative contribution items address the carbon footprint of older people as well as their perceived failure to act to arrest climate change. The perceived positive contribution items address the advocacy efforts of older people who engage in climate change mitigation and adaptation efforts, and the potential for intergenerational solidarity. Consistent domains were identified in a recent analysis of influential climate activists’ views about intergenerational relations in the context of climate change (Roy & Ayalon, 2022).

The focus on both negative and positive contributions of older people to climate change and climate action respectively is an advantage as it highlights the complex phenomenon of ageism and allows for both negative and positive aspects of ageism towards older people to co-occur (Ayalon et al., 2022a). Negative and positive ageist attitudes do not necessarily correlate. For instance, Cuddy and Fiske (2002) have identified competence and warmth as two dimensions along which different population groups are being stereotyped, with older people being seen as high on warmth, but low on competence, but other population groups being categorized differently along these two dimensions. Our findings show that perceptions of older people’s negative contribution to climate change do not necessarily negate perceptions concerning the possibility that older people could contribute to the climate change movement. Hence, people can report high levels of negative ageist attitudes but also acknowledge the potential for solidarity and the positive contribution of older people to climate action.

As expected, our study has demonstrated age differences in the perceived contribution of older people to climate change. Younger people were more likely to report negative contributions of older people to climate change, thus taking a more conflictual intergenerational stand, whereas older people were more likely to report perceived positive contributions, thus advocating for the potential of intergenerational solidarity, and perhaps attempting to deflect negative perceptions about the historic (and on-going) role of older generations in the degeneration of the planet. This is somewhat consistent with past research which has stressed the frustration of younger generations with the inaction of adults (O’brien et al., 2018). This is also consistent with past research which has found higher levels of ageism toward older people reported by younger people compared with older people (Rupp et al., 2005).

Unexpectedly, women were more likely to report the perceived negative contribution of older people in two of the three samples recruited for this study. Past research has shown higher levels of ageism among male college students compared with females (Kalavar, 2001). Consistently, two studies that examined negative and positive ageist behaviors have found that older women were more likely to report positive ageist behaviors compared with men (Cherry et al., 2016; Cherry & Palmore, 2008). However, others have found no indication of gender difference (Kalavar, 2001). Hence, although women are more likely to experience ageism (Krekula et al., 2018), research concerning gender differences in reports of ageism has been inconclusive. When it comes to climate change knowledge and attitudes, research has shown that women are more knowledgeable and more concerned about climate change (McCright, 2010). A different study, relying on data from a large sample of countries has concluded that female political representations in government are associated with lower carbon dioxide emissions (Mavisakalyan & Tarverdi, 2019). Taking these studies together, it is possible to conclude that although women have been shown to hold less ageist attitudes compared with men in some studies, they are generally more concerned about climate change and possibly more committed to climate change adaptation and mitigation efforts. This could explain the tendency of women to endorse the negative contribution of older people to climate change compared with men. However, as this finding was not consistent across the three samples, further research is required.

In the case of climate change, intergenerational issues address the temporal dimension of social justice (Page, 1999). It is the emphasis on temporal injustice which often is manifested as ageist attitudes toward older people in climate change discourse (Ayalon, 2020). Our findings show that prescriptive negative ageist attitudes toward older people are positively correlated with perceived negative contributions of older people and negatively correlated with perceived positive contributions of older people to climate action. This highlights the role of ageism in coloring intergenerational relations in the context of climate change (Ayalon et al., 2022a).

Death anxiety was correlated with beliefs concerning the negative contribution of older people to climate change, but not with beliefs concerning their positive contribution. The terror management theory (Martens et al., 2005) explains ageism towards older people as being motivated by fear of death. In order not to face their own mortality, people try to stay away from older people and devalue them. This increases one’s self-worth and sense of security and thus, serves as a means to deal with increasing anxiety brought on by reminders of one’s own inevitable mortality. Past research has argued for an association between death anxiety and climate change concerns (Budziszewska & Jonsson, 2021). The present study adds by highlighting the selective nature of death anxiety, which is directly correlated with perceptions concerning the negative contribution of older people to climate change (e.g., negative ageism) but is not correlated with perceived positive contributions of older people to climate change adaptation and mitigation efforts (e.g., positive ageism). This may perhaps be reflective of evolving public perceptions about older people and their positive contributions to society, especially advocacy for climate action.

Despite its strength, this study has several limitations that should be acknowledged. First and foremost, ageism is not a-symmetrically directed only toward older people. Younger people also experience ageism in the context of climate change and often experience situations in which their voice is not heard because of their age (Bergmann & Ossewaarde, 2020). The present study has not examined ageism directed toward younger individuals; thus, part of the picture is still missing. The samples selected are nonrepresentative and we have no data on nonresponse rate. We also did not establish the stability of the new measure over time. In addition, it is important to acknowledge the limitations of the newly constructed measure, which has demonstrated moderate fit and structural equivalence across different countries, but not metric or scalar invariance. The fact that item loadings were not equivalent across samples implies that unstandardized regression coefficients cannot be compared across different groups. We do not have convergent and divergent validity data from India, only for Australia. We also did not attempt to reframe omitted items and re-test their suitability to the new measure. Hence, we possibly limited the scope of the constructs captured by the current measure. Finally, it is important to note that although the present study and measure addressed the two poles as negative and positive ageism, some may argue that blaming older people for the current situation is justified and does not represent ageism, but rather an accurate evaluation of their inaction and responsibility for the current climate situation (Moody, 2014, 2017). Hence, it is important to note preexisting values and perceptions that have led to the development of the present measure.

Nonetheless, the newly developed measure represents a step forward in the understanding of ageism in the context of climate change. It addresses both negative and positive attitudes concerning the contribution of older people to climate change effects as well as their involvement in adaptation and mitigation efforts. The measure has demonstrated adequate validity and reliability across three different samples recruited in two different countries. This will assist in highlighting a relatively neglected area in the current climate change discourse and will potentially assist in identifying ways to improve intergenerational solidarity as part of climate change adaptation and mitigation efforts towards a world for all ages (World Health Organization, 2022). The UN Decade of Healthy Ageing has identified ageism as one of the four pillars that must be addressed in order to ensure healthy aging and healthy longevity (World Health Organization, 2021b). A more recent report concluded that a healthy climate is needed for healthy aging and healthy longevity (World Health Organization, 2022). Hence, the present study provides a direct contribution to the objectives of the UN Decade of Healthy Ageing by addressing ageism in the context of climate change in order to modify the way we think, feel, and act about age and aging to eventually live in a world for all ages.

## Supplementary Material

igad095_suppl_Supplementary_Material

## Data Availability

Data are still being analyzed and therefore are not yet available for external researchers.
