# Supplementary material for: The Perceived Contribution of Older People to Climate Change Impact, Mitigation, and Adaptation: Measurement Development and Validation
Source: Innov Aging. 2023 Sep 9;7(8):igad095. doi: 10.1093/geroni/igad095 (PMC10576513; doi:10.1093/geroni/igad095)
Supplement: igad095_suppl_Supplementary_Material [file igad095_suppl_supplementary_material.docx]

**Online Supplementary Material**

Original item poll (n=18 items)

1. Climate change affects younger people (under 18) more than older people (over age 60).
2. Climate change is a direct consequence of the unsustainable lifestyles led by older people.
3. Older people are living far too long and becoming a burden on the planet’s resources.
4. Older people’s maximum benefit to society would be to reduce their carbon footprint by withdrawing from active life after retirement.
5. Older people are selfish about accumulating wealth even though it harms the planet and the future of younger generations
6. Older leaders waste opportunities to work for the benefit of the planet
7. The youth-led global climate movement is a good way to teach older people about the impacts of climate change.
8. Older people have limited will to work for the betterment of the planet.
9. Older people have too much voting power on issues like climate change.
10. Climate change is a matter of intergenerational justice – older people of today must make sacrifices for future generations.
11. Older people are wrongly accused of destroying the planet.
12. Older people have a wealth of knowledge about sustainable living.
13. Older people can be powerful allies in the fight against climate change.
14. Older people are genuinely concerned about climate change because it affects the futures of their children and grandchildren.
15. Older people are more vulnerable to impacts of climate change than younger people
16. Many older people actively support and participate in the youth climate movement.
17. Many older leaders use their office to fight climate change for the benefit of future generations.
18. Older people can be trusted to vote for political candidates who support the fight against climate change.
